# Neural Network Development in Late Adolescents during Observation of Risk-Taking Action

**DOI:** 10.1371/journal.pone.0039527

**Published:** 2012-06-29

**Authors:** Miyuki Tamura, Yoshiya Moriguchi, Shigekazu Higuchi, Akiko Hida, Minori Enomoto, Jun Umezawa, Kazuo Mishima

**Affiliations:** 1 Faculty of Human Arts and Sciences, University of Human Arts and Sciences, Saitama, Japan; 2 Department of Psychophysiology, National Institute of Mental Health, National Center of Neurology and Psychiatry, Tokyo, Japan; 3 Department of Clinical Neuroimaging, Integrative Brain Imaging Center, National Center of Neurology and Psychiatry, Tokyo, Japan; 4 Faculty of Design, Kyushu University, Fukuoka, Japan; 5 Epidemiology and Prevention Division, Research Center for Cancer Prevention and Screening, National Cancer Center, Tokyo, Japan; University of Bologna, Italy

## Abstract

Emotional maturity and social awareness are important for adolescents, particularly college students beginning to face the challenges and risks of the adult world. However, there has been relatively little research into personality maturation and psychological development during late adolescence and the neural changes underlying this development. We investigated the correlation between psychological properties (neuroticism, extraversion, anxiety, and depression) and age among late adolescents (*n* = 25, from 18 years and 1 month to 22 years and 8 months). The results revealed that late adolescents became less neurotic, less anxious, less depressive and more extraverted as they aged. Participants then observed video clips depicting hand movements with and without a risk of harm (risk-taking or safe actions) during functional magnetic resonance imaging (fMRI). The results revealed that risk-taking actions elicited significantly stronger activation in the bilateral inferior parietal lobule, temporal visual regions (superior/middle temporal areas), and parieto-occipital visual areas (cuneus, middle occipital gyri, precuneus). We found positive correlations of age and extraversion with neural activation in the insula, middle temporal gyrus, lingual gyrus, and precuneus. We also found a negative correlation of age and anxiety with activation in the angular gyrus, precentral gyrus, and red nucleus/substantia nigra. Moreover, we found that insula activation mediated the relationship between age and extraversion. Overall, our results indicate that late adolescents become less anxious and more extraverted with age, a process involving functional neural changes in brain networks related to social cognition and emotional processing. The possible neural mechanisms of psychological and social maturation during late adolescence are discussed.

## Introduction

*Late adolescence* is a unique and important period for human development. Erikson (1994) examined the concept of *identity* in relation to late adolescence [1], [2]. Although Erikson considered identity formation to be a life-long process, he emphasized late adolescence as a key stage in his developmental theory, constituting a particular critical development period when a sense of personal and social identity becomes integrated through ‘identity crisis’. Newman and Newman (2007, 2009) redefined Erikson’s criteria regarding developmental stages, dividing adolescence into early and late adolescence, such that late adolescence (18–24) is distinguished from adolescence (12–18) [3], [4]. Erikson theorized that identity develops when young people are given a psycho-social ‘moratorium’, referring to an opportunity in which they can experiment with different social roles before making permanent commitments to an occupation, to intimate relationships, to social groups and communities, and to a philosophy of life. This ‘moratorium’ period [5] closely corresponds to ‘college age’; attending college provides students with consciousness-raising experiences to learn about themselves and others through exposure to diverse perspectives, opinions, and ways of living [6], [7], [8]. Examining psychological changes in college-age late adolescents is valuable in elucidating the ongoing process of human identity integration or maturity [9], [10], [11].

Understanding the late adolescence period is also of particular importance because dynamic psychological changes, such as human identity integration or maturity, continue throughout this period, as social and affective instabilities are overcome. Contrary to a long-held assumption that the brain is largely mature by the end of childhood, recent neuroimaging studies have provided increasing evidence that adolescence involves profound brain growth and change [12], [13]. For example, increases in white matter volume have been reported throughout childhood and adolescence, particularly in the prefrontal and parietal cortices (e.g., [14], [15], [16], [17], [18], [19], [20]. In addition, grey matter volume has been reported to increase in the prefrontal and parietal cortices during the preadolescent stage, followed by a steady decline during late adolescence [14], [21]. These findings indicate that brain development in adolescence is not linear, and that the brain volume of a typical early adolescent is measurably different to that of a typical late adolescent. In addition, these findings suggest that brain regions involved in planning, decision-making, cognitive reasoning, or controlling impulses and emotions undergo refinement through adolescence at least into a person’s twenties (i.e., late adolescence).

In addition to the morphometric studies discussed above, behavioral techniques have been used to examine brain development during late and post adolescence. For example, a behavioral study using a mentalizing task requiring theory of mind and executive function reported that social abilities like ‘theory of mind’ continue to improve from adolescence to adulthood [22], further suggesting that developmental changes continue throughout the late adolescent phase. Another study using a gambling task reported that the rate of risky choices did not significantly change between early (12–15 y.o.) and mid (15–18 y.o.) adolescence, but was significantly reduced in adulthood (25–35 y.o.) [23]. These findings indicate that a profound ‘shift’ in cognitive or emotional regulation ability during late adolescence may occur in the transition from adolescence to adulthood. Functional neuroimaging studies of mental-state attribution have reported decreases in frontal cortex activity between adolescence and adulthood [12], providing further evidence that a developmental shift occurs during late adolescence, i.e., from adolescence to adulthood. A similar discrepancy between adolescence and adulthood has been observed in the neural correlates of emotional processing. For example, in processing fearful facial expressions, adolescents were found to exhibit a strong reliance on the emotional network in the brain, while adults tended to rely more on an attentional network [24]. In addition, adults, compared with adolescents, exhibited decreased activity in the hippocampus during the encoding of negative images [25]. In accord with the studies discussed earlier, this evidence indicates that late adolescence is important as a transitional period from adolescence to adulthood, involving the maturation of emotional regulation and cognitive processing in social situations.

Several studies have reported the usefulness of a five-factor model that describes five distinct personality traits for parsing personality constructs in late adolescents [26], [27], [28]. Of the five factors, neuroticism and extraversion are of particular interest, as they are believed to be crucial for the development of healthy social interactions and to exert an over-arching influence on affect and mood. Extraversion is characterized by an increased tendency to be optimistic, and to experience positive emotions and enhanced sociability. Conversely, neuroticism is defined as an increased tendency to worry and to experience psychological distress, accompanied by negative affect and over-sensitivity to negative cues. Functional neuroimaging studies have demonstrated that task-evoked brain activity varies with neuroticism and extraversion scores in the prefrontal cortex and cingulate cortex [29], [30], [31], [32], [33]. Thus, these two personality traits are strongly associated with emotional experience and may modulate emotion-evoked brain activity [29].

Development of personality traits occurs not only in adulthood, but also in childhood and adolescence [34]. Neuroticism and extraversion are consistently included in personality models, including 3-factor and 5-factor models [35], [36], [37]. In addition, the two dimensions seem to be most related to age, educational level, and positive/negative life events from late adolescence to young adulthood [38], [39], [40]. A longitudinal study of college students reported that an increase in positive life events with age was associated with extraversion, while an increase in negative events was associated with neuroticism [40]. Another longitudinal study showed that extraversion in high school students predicted their experience of more positive life events over 4 years later in their college- or work-life, while their neuroticism predicted the experience of more negative life events [39]. These studies highlight the importance of understanding extraversion and neuroticism during late adolescence.

Moreover, a meta-analysis of 92 longitudinal studies revealed that the largest changes in personality traits occurred between ages 18 and 30, and, specifically, that late adolescents typically become more socially dominant (a facet of extraversion) and less neurotic [41]. The study indicates that late adolescence is a critical period for the development of personality traits, showing that extraversion and neuroticism are influential for late adolescents in adapting to society as they mature.

Recently, extraversion and neuroticism were also shown to impact on structural features of the prefrontal cortex in adult and elderly populations [42], [43], suggesting that extraversion and neuroticism are related to structural brain development in the earlier stages of life. Low extraversion and high neuroticism are also associated with depression, anxiety [44], and phobia [45], which are all related to psychological and psychiatric problems frequently occurring in late adolescence [45], [46]. Neuroticism also predisposes individuals to develop chronic functional pain/pain disorders [47] and mood disorders [48], [49], which are also common problems among late adolescents [50], [51]. The way in which adolescents become extraverted and less neurotic with regard to the challenging external environment during the late adolescent period is a developmental issue that has not been adequately addressed. Moreover, the neural correlates of this process are currently unclear.

In a review study examining social and emotional development during late adolescence, depressive and anxiety symptoms were found to be predictive of changes in psychosocial functioning [52]. For example, rejection sensitivity (the tendency to anxiously expect, readily perceive, and intensely react to rejection) appears to be particularly salient in late adolescence as anxiety or angry expectation [53], and was linked to a relative increase in adolescent depressive and anxiety symptoms [54]. Similarly, social anxiety was predictive of physical/psychological ‘dating aggression’ among late adolescents [55]. Moreover, healthy adolescents between 12 and 21 years old, who engaged in more extracurricular activities (i.e., participation in organized sports teams, clubs, etc.) and experienced higher quality family relationships, presented with significantly less depressive symptoms [56]. Since late adolescents face increasingly complex social situations, symptoms of depression and anxiety may be particularly damaging for the development of social competence.

In the current study, we used functional magnetic resonance imaging (fMRI) to measure neural responses elicited by the observation of actions associated with a certain risk. Moreover, we investigated the influence of developmental differences of psychological properties among late adolescents. We administered psychological questionnaires to measure anxiety, depression, neuroticism, and extraversion, all of which play a crucial developmental role in the establishment of identity during late adolescence. During fMRI scanning, participants viewed hand movements associated with a risk of harm (risk-taking actions) or no risk of harm (safe actions). This task was designed to represent common situations involving potential risks in an everyday environment, providing an index of how late adolescents are likely to cope with potential risks in their social lives in the future. Late adolescence is a challenging period characterized by pervasive social role changes across many domains [57], [58]. Salient tasks of late adolescence include goals relating to friendship, academic success, and social conduct, giving way to occupational and romantic goals as late adolescents move into young adulthood [9]. The large number of changes faced in late adolescence make it an unstable time, but also reflects the explorations that take place during the late adolescent years. Many of the changes made by late adolescents are for the purpose of some new period of exploration, in love, work, or education. In accord with this notion, it is possible that a late adolescent’s level of tolerance of risk-taking actions may become entrenched as they are frequently confronted with challenging social situations during late adolescence. Here, we postulated that action observation of risk-taking would be a developmental indicator of motivation to overcome a broad range of difficulties in the world. Although teenagers are generally regarded as engaging in more ‘risky behavior’, such as binge drinking, cigarette smoking, having casual sex partners, violence and other criminal behavior etc [59], [60], [61], it should be noted that in this study we do not use the term ‘risk-taking’ to refer to a tendency to such a ‘risky behavior’. Rather, we use ‘risk-taking’ to refer to a more positive concept, whereby adolescents confront and manage the difficulties facing them.

Although this is an exploratory analysis, we hypothesized that late adolescents will become less anxious, less neurotic, less depressive, and more extraverted as they age, measured by the correlation between age and questionnaire scores, and that neural responses to the observation of risk-taking actions will be modified as adolescents become tolerant of risks in the external environment. Furthermore, we hypothesized that the developmental aspects of their tolerance to the external environment (measured as the correlation between questionnaire scores and age) will be mediated by changes of neural circuitry. These changes may involve the limbic or paralimbic systems (e.g., insula) or brainstem, which are central to the processing of affective information. In addition, the changes may also affect the prefrontal areas, which are important for emotional regulation. Activity in the anterior insula has been found to be associated with empathic maturity during the observation of emotional expressions among children [62]. This finding supports the notion that the insula is relevant to social functioning in everyday life.

## Materials and Methods

### Participants

Twenty-five participants in their late adolescence took part in this experiment (12 females and 13 males, from 18 years and 1 month to 22 years and 8 months, *M* ± *SD* years: 20.60±1.09). All participants were undergraduate students and had normal or corrected-to-normal vision. All participants were right-handed (lateralization quotient for the right side of more than 90%) as assessed by the Edinburgh Handedness Inventory [62]. Written informed consent was given before participation in the study, which was specifically approved by the Institutional Ethical Review Board of the National Center of Neurology and Psychiatry, Japan. All participants were screened to rule out head trauma, the use of medication, history of neurological or psychiatric disorders, and other serious medical conditions.

### Image Acquisition

Images were acquired using a 1.5 T Magnetom Vision plus MRI scanner (Siemens, Erlangen, Germany). We acquired a unique high-resolution structural image (T1-weigthed anatomical images; 3D MP-RAGE sequence, repetition time; TR  = 11.4 ms, echo time; TE  = 4.4 ms, flip angle  = 15°, 256×256 matrix, slice thickness 1.25 mm) with 144 sagittal slices after the functional runs. Each functional run involved the acquisition functional echo-planar imaging (EPI) volumes (gradient-echo, TR  = 3,000 ms, TE  = 40 ms, field of view; FOV  = 192 mm, flip angle  = 90°, 64×64 matrix, slice thickness 3.5 mm), each with 36 interleaved slices approximately parallel to the anterior commissure-posterior commissure line. Stimuli were displayed on a screen positioned at the rear of the scanner, which the participant could comfortably see through a mirror mounted on the standard head coil.

### Psychological Measures

Prior to the fMRI session, participants completed the Maudsley Personality Inventory (MPI) [63], [64], Spielberger State-Trait Anxiety Inventory (STAI) [65], [66], and the Self-rating Depression Scale (SDS) [67], [68]. The MPI consists of 80 items, each assessing a constellation of traits, also providing a measure of personality along the neuroticism and extraversion scales. The STAI-trait is a self-report instrument of the longstanding quality of trait anxiety. The STAI-trait consists of 20 items, and high total scores indicate more trait anxiety. The SDS was developed as a self-administered measure of depression severity, with higher scores indicating more severe depression. The 20 items of the scale address each of the four most commonly found characteristics of depression: pervasive effects, physiological equivalence, other disturbances, and psychomotor activities.

### Action Observation Stimuli and Procedure

**Figure 1:**
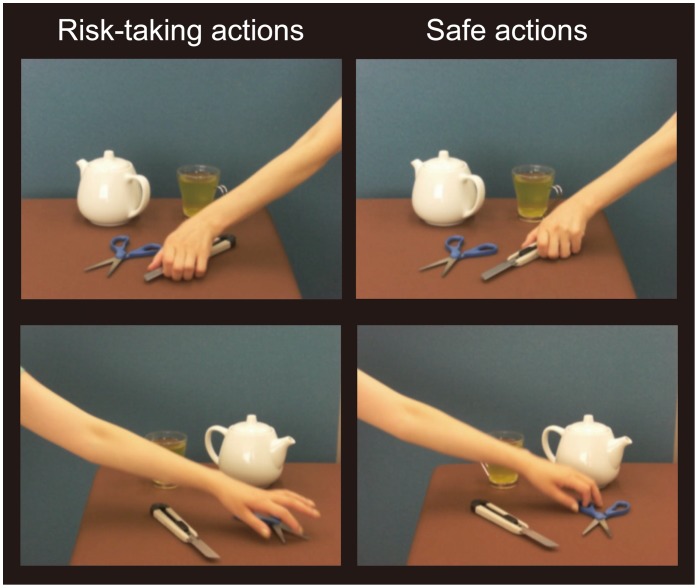
Examples of movies (risk-taking and safe).

The action observation experiment involved two types of video clips, ‘risk-taking’ (video of a person’s hand performing an action with clear potential for causing harm to oneself) and ‘safe’ (showing a person’s hand performing an action with no clear danger) (Figure 1). The stimuli are described in detail in Table 1. It should be noted that the video clips were not created to convey the meaning of problematic behaviors among adolescents, such as using drugs or alcohol, driving drunk, smoking, unprotected sex, or other offensive or criminal activities. Rather, the video clips presented participants with situations involving common risks that most people are exposed to in everyday life. Each functional run began and ended with the presentation of a white fixation dot for 9 s. Between these two fixation periods, video clips from the two conditions were presented in alternating 21 s blocks. Each block consisted of a 21 s video clip, and each baseline consisted of a 9 s fixation period. Each block contained videos depicting three different risk-taking actions, or three different safe actions. Half of the blocks showed people performing actions from the right of the screen, and half from the left. Participants completed 10 blocks of each condition during a single scan. The order of presentation of the stimuli was determined according to an optimized random sequence for each block. The brightness of the screen, the intensity of contrast (luminance contrast and texture contrast), the velocity of hand actions, and the representation of objects were equalized for all task/control video clips. The total duration of the risk-taking video clip equaled the duration of the safe video clip (the length of each video clip was 7.0 s). Three functional runs, lasting 10 minutes each, were collected for each participant. The hand movements of participants were monitored by direct visual inspection and video-monitoring from the back of the fMRI tunnel. No visible movements were noted during the presentation of the experimental stimuli.

**Table 1:** Experimental conditions and Visual Analog Scale (VAS) score (100-0) for each condition.

Action description	Risk-taking-Safety	Anxiety-Comfort
*Risk-taking actions*		
Grasping and placing the blade of the box-cutter knife	80.2±17.9	83.4±10.3
Grasping and placing the clipper blade of the scissors	80.3±9.0	76.5±12.0
Grasping and placing the brimming cup	39.2±18.7	31.0±25.1
*Safe actions*		
Grasping and placing the handle of the box-cutter knife	36.7±25.1	36.6±24.5
Grasping and placing the scissors	35.0±21.0	36.1±20.6
Grasping and placing the pots	21.7±21.4	20.8±18.5

Data are presented as mean ± standard deviation.

### Behavioral Measures

After the scanning procedure, each participant was shown the video clips again, and asked to answer specific questions related to them. To test subjective evaluations of the degree of risk, participants were asked to rate their subjective experience of the video clips using a visual analog scale (VAS) (1) from feelings of risk to safety (i.e. to what extent did you feel alarmed while watching the video clip?) and (2) from feelings of anxiety to comfort (i.e. how anxious did you feel while watching the video clip?).

### fMRI Data Analysis

Image processing was carried out using statistical parametric mapping software (SPM8, the Wellcome Trust Centre for Neuroimaging, London, UK). The functional time series was motion corrected, slice timing corrected and smoothed with a Gaussian kernel of 8 mm full-width at half-maximum. The corresponding high-resolution structural image (the T1 image as the source image) was registered to the first EPI image as the reference image. The co-registered structural image was then transformed into standard anatomical space using the Montreal Neurological Institute structural template (MNI 152). These parameters were used to normalize all functional images. Following preprocessing, ‘Risk-taking’ and ‘Safe’ condition, and motion parameters (six realignment parameters) were entered as regressors. A high-pass filter (hpf) of 128 sec was also applied as regressors. The risk-taking and safe blocks were convolved with a hemodynamic response function without derivatives, and modeled as 21 s boxcar regressors. The 9 s fixation periods were modeled as implicit baseline blocks. Next, a first fixed level of analysis was computed subject-wise using the general linear model. The following T-contrasts were estimated: risk-taking, safe, risk-taking vs. safe, and safe vs. risk-taking.

### Neural Response to Observation of Risk-taking versus Safe Actions

To test our hypothesis that activation in areas related to action observation would be significantly enhanced when actions involved risk-taking, we compared activation in each condition using linear contrasts (risk-taking versus safe and safe versus risk-taking). The resulting set of voxel values for each contrast constituted a statistical parametric map of the *t* statistic SPM(*t*). Anatomical localization was performed in MNI coordinates. Talairach coordinates (Talairach Daemon, www.talairach.org/daemon.html) were used for anatomical localization to be compared with Brodmann areas [69]. Significant activations were defined using a lenient height-threshold of *p*<0.001, uncorrected, and an extent threshold of *k* = 10 (voxels), to reduce the risk of false negatives. Our use of cluster size thresholding combined with uncorrected *p* values was intended to adequately control for the prevalence of false positives [70]. This threshold suffices to eliminate speculation that effects observed in the primary parametric analysis are an artifact due to non-specific reductions in BOLD signal.

### Neural Activity Associated with Age and Psychological Measures

This analysis aimed to reveal the brain regions in which activity mediates the age-related change of psychological properties that are essential in individual maturity during late adolescence. In a second-level random-effect analysis, participant’s imaging data were regressed with psychological scores (neuroticism, extraversion, anxiety and depression) and age with a multiple regression analysis. The correlation map of neural responses to risk-taking action (the main SPM(*t*) contrast of risk-taking vs. safe actions) with age and the correlation map of the same neural response with each of the psychological scores were calculated separately. A conjunction analysis was then performed to show overlapping areas of the two correlational maps: an age-related activation map and a personality-related map.

Parameter estimates were extracted from the regions surviving the conjunction analyses that tested for statistical mediation using the INDIRECT macro for SPSS (http://www.afhayes.com/) [71], [72], [73], [74]. According to Baron and Kenny (1986), four steps are required to establish that neural activity in a particular region mediates the relation between age and psychological properties: (1) showing that age is associated with psychological properties; (2) showing that age is associated with neural activity in the region; (3) showing that neural activity in the region predicts psychological properties when controlling for age; and (4) showing that the relation between age and psychological properties is reduced when controlling for neural activity in the region. For a sample of 20–80 participants, statisticians recommend the use of bootstrapping methods for testing the statistical significance of mediation (rather than the Sobel test, which is appropriate for larger samples; [71], [72]. The current study used the bootstrapping approach outlined by Shrout and Bolger (2002), which provides a mean estimate of the indirect effect (i.e., the path through the mediator) and the associated 95% confidence interval. A confidence interval that does not contain zero indicates statistically significant mediation (*p*<0.05). Cook’s distance metric was used to test whether data from a few individuals unduly influenced the strength of the bivariate relationships. A value (age, psychological property, and neural activity) greater than 1 for a data point represents a statistical outlier [75]. No point had a Cook’s distance greater than 0.5, indicating that none of the correlations were dependent on statistical outliers.

## Results

### Behavioral Measures

Table 2 shows descriptive features of psychological measurements (neuroticism, extraversion, anxiety-trait, and depression scores) and the correlation coefficients between these scores and age. The scores of neuroticism, anxiety, and depression were negatively correlated with age (*r*_(23)_ = −0.49, −0.56, and −0.54, respectively), and extraversion scores were positively correlated with age (*r*_(23)_ = 0.44), suggesting that the late adolescents became less neurotic, less anxious, less depressive, and more extraverted with age. These scores were used for the multiple regression analysis of neural responses, regressed with age and psychological variables.

**Table 2:** Psychological assessment scores and correlation coefficients with age for each score.

Psychologicalmeasurement	Score	Correlationcoefficient with age
Maudsley Personality Inventory (MPI)		
Neuroticism	22.7±11.1	−0.49*
Extraversion	33. 6±10.3	0.44*
State-Trait Anxiety Inventory (STAI-trait)		
Anxiety-trait	44.9±10.6	−0.56**
Self-rating Depression Scale (SDS)		
Depression	37.7±9.1	−0.54**

Data are presented as mean ± standard deviation.

Spearman’s *ρ*: *p<0.05; **p<0.01.

Table 3 shows the scores of subjective ratings using the visual analog scale for each video clip. To test the efficiency of the categorization of the stimuli in terms of risks, the scores of 1) participants’ subjective levels of risk-taking and 2) the extent to which they felt anxious when observing the stimuli were compared between the two within-participant categories (risk-taking and safe actions in video clips). The results indicated that participants experienced significantly stronger feelings of risk-taking and anxiety during the observation of risk-taking compared with safe actions (*F*_(1, 24)_ = 151.16, *p*<0.001, and *F*_(1, 24)_ = 93.73, *p*<0.001, respectively, using repeated-measures ANOVA). To validate the video clip task used in this study, we calculated the correlation coefficients of the subjective ratings of the feeling (“risky” and “anxious”) induced by the actions in the video clips with psychological assessments (correlation coefficients *r* are provided in the Table 3). The VAS scores of feeling “risky” about Risk-taking actions were positively correlated with anxiety (*r*_(23)_ = 0.41), and negatively correlated with extraversion (*r*_(23)_ = −0.42). VAS scores for feeling “anxiety” about Risk-taking actions were positively correlated with neuroticism and anxiety (*r*_(23)_ = 0.44 and 0.44, respectively), and negatively correlated with extraversion (*r*_(23)_ = −0.40). In contrast, VAS ratings for safe actions were not correlated with any psychological assessment. These results indicate that the video task in this study (the observation of risk-taking action) provides a suitable measure of the psychological factors of interest in this study, and can be used to examine important developmental processes in the late adolescence period.

**Table 3:** Correlation coefficients between psychological assessments and subjective ratings (“risky” and “anxiety”) about the actions in video clips.

	Neuroticism	Extraversion	Anxiety	Depression
*Risk-taking actions*				
“risky”	0.32	−0.42*	0.41*	0.35
“anxiety”	0.44*	−0.40*	0.44*	0.36
*Safe actions*				
“risky”	0.03	−0.27	0.19	0.30
“anxiety”	0.10	−0.09	0.14	0.35

Bold type*: *p*<0.05.

### Neural Response to Risk-taking versus Safe Actions

**Figure 2:**
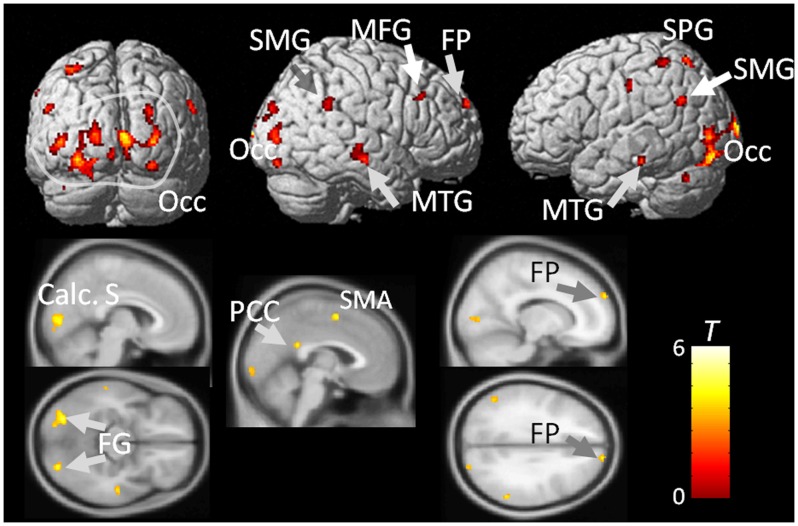
Brain images of neural activity in response to the observation of the object-related hand movement task for risk-taking actions vs. safe actions. Statistical threshold for illustrating the clusters was *p*<0.001 uncorrected. The bar on the right shows the range of *t* scores for statistical parametric mapping. Calc. S, calcarine sulcus; FG, fusiform gyrus; FP, frontal pole; MFG, middle frontal gyrus; MTG, middle temporal gyrus; Occ, occipital cortex; SMA, supplementary motor area; SMG, supramarginal gyrus; SPG, superior parietal gyrus.

We compared the neural activation elicited by observing risk-taking versus safe actions across the whole brain (Figure 2, Table S1). The results revealed that risk-taking actions elicited significantly stronger activation, mainly in the bilateral middle frontal gyrus (BA9/10), superior frontal gyrus/frontal pole (BA8/10), supramarginal gyrus (BA39/40), inferior parietal lobule (BA40), superior temporal gyri (BA22/39), middle occipital gyri (BA18/19), and cuneus (including the calcarine sulcus) (BA17/18/19) compared with safe actions. Additional areas of significant activation were also found in the left middle temporal gyrus (BA21/22), medial frontal gyri (supplementary motor area) (BA6), superior parietal gyrus/lobule (BA7), precentral gyrus (BA6), posterior cingulate (BA23), fusiform gyrus (BA37), lingual gyrus (BA17), insula (BA13), and declive. In the right hemisphere, we observed significant activation in the precuneus (BA7) and postcentral gyrus (BA2). No regions exhibited greater activation while viewing safe actions compared with risk-taking actions associated with risk.

### Neural Responses Mediating Relationship between Age and Psychological Properties

Finally, we performed a conjunction analysis to reveal common brain activity that correlated both with age and psychological scores (neuroticism, extraversion, anxiety, and depression) (Table 4). A positive correlation between age and neural response to observation of risk-taking action [*r*_age(+)(23)_, *p*<.001] and a positive correlation with extraversion [*r*_e(+)(23)_, *p*<.001] were found to overlap in the insula [BA13, *r*_age(+)_ = 0.70, *r*_e(+)_ = 0.76], middle temporal gyrus [BA22, *r*_age(+)_ = 0.68, *r*_e(+)_ = 0.70, ], and precuneus [BA19, *r*_age(+)_ = 0.67, *r*_e(+)_ = 0.67]. A positive correlation with age [*r*_age(+)(23)_, *p*<.001] and a negative correlation with anxiety [*r*_anx(-)(23)_, *p*<.001] were found to overlap in the angular gyrus/supramarginal gyrus [BA39, *r*_age(+)_ = 0.68, *r*_anx(-)_ = −0.79, ], precentral gyrus [BA6, *r*_age(+)_ = 0.67, *r*_anx(-)_ = −0.73, ], and red nucleus/substantia nigra [*r*_age(+)_ = 0.66, *r*_anx(-)_ = −0.79]. Parameter estimates were extracted from the regions surviving the conjunction analysis. The parameter estimates were used in a series of analyses testing for statistical mediation.

**Table 4:** Brain regions mediating association between psychological measurement and age.

		MNI				Cluster
Anatomical region	BA	*x*	*y*	*z*	*T*	*k*
***Neuroticism-Age***						
*no mediation*						
***Extraversion-Age***						
*Positive correlation with age*						
Insula	13	−40	−10	20	4.55	32
Middle temporal gyrus	22	−58	−34	−2	4.45	23
Precuneus	19	36	−74	36	4.27	19
*Negative correlation with age*						
*no mediation*						
***Anxiety-Age***						
*Positive correlation with age*						
*no mediation*						
*Negative correlation with age*						
Angular gyrus/Supramarginal gyrus		40	−62	34	4.39	23
Precentral gyrus	6	16	−20	64	4.23	18
Red nucleus, Substantia nigra		8	−22	−8	4.14	16
***Depression-Age***						
*no mediation*						

Statistical threshold: *p*<0.001 uncorrected, *k* = 10.

MNI refers to Montreal Neurological Institute coordinates; BA refers to putative Brodmann Area; L and R refer to left and right hemispheres.

**Figure 3:**
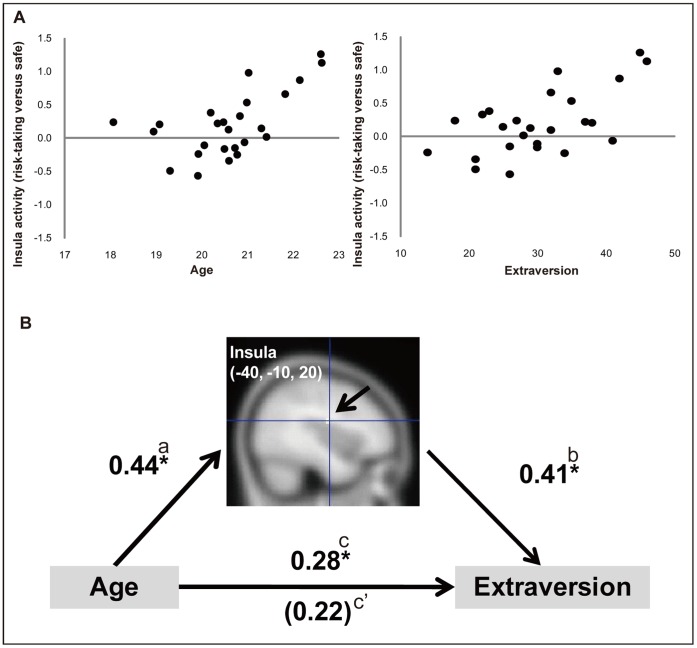
(A) Scatterplots of associations between insula activity (BA13, peak in MNI space: −40 −10 20) and age for the peak of the clusters surviving conjunction analysis with an independent regression of extraversion. Left panel: association between age and insula activity (*r* = 0.60, *p*<0.01). Right panel: association between extraversion and insula activity (*r* = 0.61, *p*<0.01). (B) Brain regions that mediated the relationship between age and extraversion. Parameter estimates (risk-taking > safe contrast) extracted at the region identified by conjunction analyses were independently regressed by age and psychological properties. Mediation tests were based on methods described by Shrout and Bolger (2002) and Baron and Kenny (1986). (a) Regression slope of age predicting neural activity; (b) regression slope of neural activity predicting extraversion, controlling for age; (c) regression slope of age predicting extraversion; (c’) regression slope of age predicting extraversion, controlling for neural activity. Bootstrapping was used to estimate indirect effects (Shrout & Bolger, 2002; see also Preacher & Hayes, 2004). A confidence interval that does not overlap with zero indicates statistically significant mediation. *Indicates significant difference from zero, *p*<0.05. Coordinates are given in MNI space.

As shown in Figure 3, insula activation (BA13) mediated the relation between age and extraversion. This insula activation was positively associated with age and extraversion (age: *β* = 0.44; extraversion controlling for age: *β* = 0.41). The relationship between age and extraversion (*β* = 0.28) was reduced when controlling for activity in the insula (*β* = 0.22). Bootstrapping revealed that the insula significantly mediated the relation between age and extraversion (mean indirect effect  = 5.35, 95% confidence interval ranging from 0.37 to 11.78). The results indicate that, as late adolescents’ age, their neural responses to the observation of risk-taking actions increase in the insula and middle temporal, lingual, and precuneus areas. These changes were related to developmental changes of the participants’ psychological properties, such as increased extraversion. In particular, the insula significantly mediated the relation between age and extraversion. Also, age-related increases of neural activation in the angular gyrus, precentral gyrus, and red nucleus (and substantia nigra) were found to contribute to decreasing anxiety with age.

## Discussion

The primary question motivating the present study was whether late adolescents become more extraverted, less neurotic, and less anxious as they age, and whether such changes might reflect increased tolerance to the challenges of the adult world. In addition, we also sought to test how brain function reflects developmental changes occurring in late adolescent psychology.

The results revealed several major findings. First, we observed significant correlations between age and scores on psychological parameters that have been hypothesized to play central roles in the development of the late adolescent mind: younger people tended to become more extraverted and less neurotic, less anxious and less depressive with age during late adolescence, even within the small age range in our sample. Levels of neuroticism have been previously reported to decline with age until around age 80 [76]. However, extroversion has also been found to decline with age [77]. Reports of the correlation of age with depression and anxiety are, however, inconsistent. While some studies have reported no age-related difference on the SDS and STAI among students aged 18–28 [78], another study reported correlations of anxiety and depression with age in a population with a mean age of 33.0 years [79]. To date, there has been no study reporting detailed changes of these psychological properties within a small age range in late adolescence. The robust association of age with neuroticism, extraversion, anxiety, and depression in our study indicates that the late adolescent mind undergoes dramatic changes within a short period of time. These changes appear to be unique and distinct from psychological changes that occur across the longer lifespan. Future studies with larger samples will be necessary for elucidating the nature of these dramatic changes in late adolescence.

Analysis of the “risk vs. safe” contrast revealed neural activation in areas broadly associated with action recognition. These results indicate that observing risk-taking compared with safe actions in our task elicited neural activation in; 1) occipital visual areas including the calcarine sulcus and fusiform gyrus, which are related to lower-level processing of visual information and sending output information to the action recognition network; 2) the bilateral superior and inferior parietal regions, which have been implicated in action recognition and representation [80]; 3) the posterior middle temporal gyrus, which is located in the middle of the ventral pathway and serves as a central node in the association of actions and meanings [81]; 4) the supramarginal gyrus or inferior parietal area, widely considered to be homologous to the monkey parietal mirror neuron system, which is critical for encoding and recognition of gestures such as object–related postures and movements [81]; 5) the posterior cingulate area associated with human awareness, self-reflection [82], and memory retrieval [83], [84] etc. and 6) the frontal pole (superior frontal gyrus), which has been implicated in retrospective monitoring of observed actions that affect one’s future actions [85], [86]. Therefore the results suggest that the risk content of the observed action in the video clips enhanced the broad spectrum of visually-guided action recognition processing; the network of visual input and its processing appear to encode the meaning of the observed action and even the reflective or retrospective monitoring of the action’s outcomes. As a result, the risk-related content of the action enhanced a broad network associated with action recognition. This finding indicates that risk-taking situations may increase cognitive load in the entire action recognition system, commanding more attention to the actions shown in the video clips. One possible explanation for the result is that risky actions are relatively unusual and involve novelty, which may cause more brain activation. In the present study, however, it is unlikely that factors related to novelty exerted a substantial effect on brain activation, because the situations depicted in the video clips were not unusual. Rather, the videos depicted common situations that often occur in everyday life. In addition, the degree of novelty of the experimental stimuli was controlled using control video clips. Although we did not observe strong activation in affect-related brain areas, another explanation is that the present results reflect some affective impact on brain activation related to risk-taking context. For example, activation in the mirror neuron system while observing action can be enhanced by motivational and affective aspects of the observed action, (e.g., [87]). Thus the action recognition system may be modulated by the affective context of the risk-taking actions of observed actions.

Alternatively, processing risky actions may require more cognitive resources, involving the estimation and monitoring of possible outcomes of observed actions (see [86]. This notion is in accord with our current finding that the frontopolar region was more engaged while observing risk-taking compared with safe actions. In addition, a previous study reported that the superior part of the frontal polar area exhibited stronger activation when participants thought about the future compared with when they thought about the past [85]. Moreover, human lesion studies have indicated that the frontal polar area may be involved in generating insights into one’s future [88], [89], [90]. On the basis of the current findings, taken together with previous evidence, we hypothesize that risk-taking actions require more cognitive resources to process, involving the estimation of action outcomes resulting in stronger activation in the frontopolar cortex.

The posterior cingulate cortex (PCC) was also activated by risk-taking vs. safe actions. This region may be another center for risk-related brain activity, which has been suggested by previous animal studies. For example, it has been reported that PCC activation in monkeys is sensitive to risk in decision making tasks [91]. In addition, the PCC is reported to exhibit activation when monkeys make risky choices, and to become more active with greater perceived risk [92]. These reports are consistent with the present finding that the observation of risk-taking actions (compared to safe ones) activated the PCC. The PCC is reciprocally connected to parietal areas (action recognition network) and receives feedback input from the prefrontal cortex (see the review in [93]), which is involved in estimating the possible outcomes of action. Activation in this area may have exhibited risk-related sensitivity, together with the other regions listed above.

The main finding in the current study was that neural activation during the observation of risk-taking (compared with safe) actions was correlated with age and some psychological measures, especially, extraversion and anxiety. These results indicate that these psychological properties have important developmental components during late adolescence, and that these developmental changes are represented by brain activation related to the observation of risk-taking actions in the insula and the parietal-temporal-occipital association area (middle temporal gyrus, lingual gyrus, and precuneus). Furthermore, we found that the insula significantly ‘mediated’ the relationship between age and extraversion.

The insula is a multifunctional cortical region involved in emotional processing [94], [95], [96], [97], [98], [99], speech-motor function [100], [101], [102], [103], [104], [105], aversive experience [106], both physical (i.e. visceral and somatic pain) and emotional (i.e. affect and mood) experience [94], [96], [107], conscious awareness (interoceptive awareness) [108], and emotional awareness [109]. The insula also plays a critical role in the processing of risk-taking during decision-making [110], suggesting that insula activity may also be modified by the risk-taking context of the observed action in our study.

The present finding of a correlation between insula activity and extraversion is consistent with the results of a previous positron emission tomography (PET) study [30] showing that the blood flow of the insula cortex was correlated with extraversion. Introversion (low extraversion) is associated with anxiety through increased limbic activation (in the insular cortex and amygdala), and is affected by genetic factors [111]. A morphometric study also revealed that extraversion correlated positively with gray matter volume of the insula [112]. Importantly, a previous fMRI study [113] revealed that extraversion correlates with neural responses to *positive* word stimuli in the bilateral insula. It is possible that, although the insula response to observing risk-taking actions may reflect an elevated alertness to the risk of harm in the environment, the participants did not experience substantial negative affect, as would be the case if they suffered from severe neuroticism or anxiety/depression. Rather, participants may have been receptive to the new challenges evoked by the task, which could result in the relationship between insula activity and a personality trait relating to a more positive aspect of affect, i.e., extraversion. Although insula activity reflects the processing of arousal or novelty [114], [115], this may be accompanied by positive affect to some extent.

While insula activity is related to extraversion, the insula has also been found to exhibit developmental changes [116], [117]. A neuroimaging study revealed increased activation in the insula with age in response to a risk-taking task [116]. Studies of individuals with clinical or developmental disorders consistently show insular morphometric changes, such as gyrification [117] and reduction of the insular volume in Williams syndrome [117], which further suggests developmental changes in the insula. The current finding of an age-related increase in insula activation may also support the notion that the level of affective (particularly positively-valenced) engagement in risk-taking action increases with age in late adolescence. Overall, the current results, which show a link between age and insula activity as well as a link between insula activity and extraversion, suggest that insula activity may ‘mediate’ the development of extraversion.

Our results also revealed that activity in the posteromedial parietal cortex (including the precuneus) correlated positively with extraversion. Gamma et al., (2000) report a similar positive correlation between extraversion and rCBF in the precuneus, consistent with the current findings [118]. Extraversion is related to the active seeking of social or interpersonal engagement, and the precuneus is also related to processing social information and estimating interpersonal relationships. For example, the precuneus is activated during ‘forgivability’ judgments in social scenarios [119] and in the attribution of emotions to the self and others [120]. Moreover, a number of studies have identified that the precuneus is modulated by agency and intentions in action/movement recognition [121], [122], and moral judgments [123]. The precuneus, in parallel with its social functions, shows age-related changes of brain activity during theory of mind tasks [124], [125]. The correlation between extraversion and activity in the precuneus, with its age-related changes, suggests that increased precuneus activation may mediate the process of late adolescents becoming increasingly extraverted and exhibiting improved social functioning with age.

In the current study, as late adolescents aged, activity in the angular gyrus and precentral gyrus increased, and was negatively correlated with anxiety. In a previous fMRI study [126], participants were told that an electrodermal stimulation could occur at any time (“threat”) or that no stimulation would occurs (“safe”). The results revealed stronger activity in the “safe” condition than in the “threat” condition in the angular gyrus and precentral gyrus. Thus, these regions may reflect enhanced perceptions of safety, consistent with the current finding of a negative correlation between the activity in these two regions and the level of anxiety. Simmons et al. [127] also reported reduced activity in the posterior superior temporal cortex adjacent to the angular gyrus in anxiety-prone participants compared with control participants during the observation of aversive images. Moreover, the more repetitively the emotional facial pictures were presented the stronger the neural activity was in the angular/posterior superior temporal gyrus and precentral gyrus, such that the activation in these areas correlated with participants becoming habituated to the emotional stimuli and becoming less anxious [128]. The precentral gyrus has dense connections with the angular gyrus [129], and the angular gyrus and precentral gyrus have been reported to exhibit coactivation during a range of cognitive tasks, including lexical [130], [131] and calculation tasks [132]. These findings suggest the existence of a network involving these two areas. Moreover, both the angular gyrus and precentral gyrus are included in the default-mode network (DMN), a prominent large-scale brain network that exhibits strong activity during the resting state and deactivation during cognitively demanding tasks [133], [134]. We propose that maturation of a network including the angular gyrus and precentral gyrus may play an important role in stabilizing affective states during late adolescence.

Several limitations of the current study should be considered. First, the sample size was relatively small for studying personality-related factors. This may have reduced the statistical power of our analysis, potentially influencing the results. Additional studies with larger sample sizes and more detailed longitudinal behavioral and cognitive testing focusing on the developmental aspects of personality are required to verify these novel findings. Second, our mediation analysis design, consisting of three variables, may have omitted many other variables that influence both insula response and extraversion in the same direction, potentially resulting in a positive bias in the results. For example, it has been reported that empathic ability is correlated with insula activity [135]. In addition, some evidence suggests that empathic individuals are more likely to be extraverted [136]. Moreover, an independent variable can have multiple mediators, which would have been omitted in this design. These potential confounds are likely to affect mediators and dependent variables in the same way. Future studies should take into account other potential mediators. Third, the conjunction analysis shown in Supplementary Table 1 may contain false positives, meaning that the results should be corrected for multiple comparisons (e.g. Bonferroni correction). Applying the Bonferroni correction would increase the likelihood of false negatives, however. Since the current study is an exploratory examination, it may be appropriate to consider the statistical significances of conjunction analyses as preliminary values at this point. Additional studies using the Bonferroni correction will be needed to more rigorously test our hypothesis. Fourth, we found a negative correlation of age and anxiety with activation in the red nucleus/substantia nigra, but no significant effect of the main contrast (risk-taking vs. safe; in Supplementary Table 1). The substantia nigra contains dopamine-containing neurons [137], [138]. Moreover, the sequence planning and timing-related motor functions in the substantia nigra indicate dopaminergic gating of motor sequences [139], [140]. Future studies will be required to investigate this issue in more detail. Fifth, as shown in Table 3, multiple correlation analyses were computed between psychological measurements. This may have led to significant effects due to chance in each correlation analysis, such as correlation coefficients between the subjective ratings of the two kinds of video and the four psychological measurements. Adopting a more conservative corrected alpha level, however, would increase the likelihood of false negative results. In the current study, we adopted a thresholding method (*p*<0.001, uncorrected, with 10 contiguous voxels) that was initially proposed more than a decade ago [70] and has been used in many fMRI studies. A number of alternative methods of correction for multiple comparisons currently exist (e.g., [141], [142]), and the thresholding method in our study is not the only one available. It is important to note that the present correlational analysis constitutes an exploratory finding. Future studies will be required to test whether the current data can be replicated.

Overall, our findings indicate that late adolescents become less neurotic, less anxious, less depressive, and more extraverted as they age. These changes are associated with activity in brain regions related to social cognition and emotional processing.

## Supporting Information

Table S1 **Regions that demonstrate significant hemodynamic signal changes during the observation of risk-taking vs. safe actions.**
(DOC)
