# Supplementary material for: Neural Network Development in Late Adolescents during Observation of Risk-Taking Action
Source: PLoS One. 2012 Jun 29;7(6):e39527. doi: 10.1371/journal.pone.0039527 (PMC3387168; doi:10.1371/journal.pone.0039527)
Supplement: Table S1 — Regions that demonstrate significant hemodynamic signal changes during the observation of risk-taking vs. safe actions. (DOC) [file pone.0039527.s001.doc]

Supplementary Table 1. Regions that demonstrate significant hemodynamic signal changes during the observation of risk-taking vs. safe actions.

|  |  |  | MNI |  |  | Cluster |
| --- | --- | --- | --- | --- | --- | --- |
| Anatomical region | BA | *x* | *y* | *z* | *T* | *k* |
| Cuneus | 18 | -18 | -106 | 10 | 6.80 | 428 |
|  |  | -28 | -80 | -10 | 5.85 |  |
|  |  | -22 | -78 | -16 | 5.45 |  |
| Middle occipital gyrus | 19 | -42 | -82 | 4 | 5.68 | 101 |
| Middle temporal gyrus | 21 | -62 | -30 | -18 | 4.96 | 24 |
| Medial frontal gyrus (supplementary motor area) | 6 | -2 | 0 | 56 | 4.88 | 43 |
| Middle occipital gyrus | 18 | 28 | -86 | -14 | 4.87 | 44 |
| Cuneus | 17 | 6 | -84 | 4 | 4.83 | 331 |
|  |  | 30 | -88 | 6 | 4.82 |  |
|  |  | 8 | -90 | -4 | 3.85 |  |
| Superior temporal gyrus | 22 | 52 | -18 | -12 | 4.81 | 137 |
|  |  | 48 | -28 | -10 | 4.62 |  |
|  |  | 52 | -22 | 0 | 4.44 |  |
| Posterior cingulate | 23 | -6 | -42 | 22 | 4.62 | 45 |
| Superior parietal lobule / Superior parietal gyrus | 7 | -32 | -66 | 62 | 4.62 | 41 |
| Superior frontal gyrus/ Frontal pole | 8 | -16 | 48 | 46 | 4.59 | 15 |
| Superior frontal gyrus / Frontal pole | 10 | 14 | 62 | 30 | 4.55 | 31 |
| Insula | 13 | -42 | 0 | 16 | 4.51 | 20 |
| Fusiform gyrus | 37 | -38 | -64 | -30 | 4.50 | 27 |
| Inferior parietal lobule / Supramarginal gyrus | 40 | 58 | -46 | 32 | 4.46 | 42 |
| Postcentral gyrus | 2 | 36 | -32 | 38 | 4.44 | 17 |
| Precentral gyrus | 6 | -62 | -20 | 44 | 4.33 | 24 |
| Middle frontal gyrus | 10 | -36 | 42 | 14 | 4.27 | 15 |
| Middle temporal gyrus | 22 | -48 | -50 | -4 | 4.19 | 12 |
| Middle frontal gyrus | 9 | 52 | 26 | 36 | 4.13 | 28 |
| Declive |  | -16 | -64 | -28 | 4.13 | 38 |
| Superior temporal gyrus / Supramarginal gyrus | 39 | -50 | -62 | 30 | 4.12 | 39 |
| Inferior parietal lobule | 40 | -36 | -48 | 60 | 4.11 | 33 |
| Cuneus | 19 | 26 | -88 | 28 | 4.11 | 50 |
| Cuneus | 18 | 18 | -102 | 0 | 4.05 | 13 |
| Precuneus | 7 | 28 | -54 | 50 | 4.02 | 14 |
| Inferior parietal lobule/ Supramarginal gyrus | 40 | 50 | -40 | 62 | 3.99 | 12 |
| Superior parietal lobule | 7 | -28 | -68 | 48 | 3.95 | 10 |
| Lingual gyrus | 17 | -4 | -96 | -8 | 3.92 | 29 |

Statistical threshold: *p* < 0.001 uncorrected, *t* = 3.47.

MNI refers to Montreal Neurological Institute coordinates; BA refers to putative Brodmann Area; L and R refer to left and right hemispheres.
